# Genetical and Morphological Identification of *Prosthogonimus pellucidus* (Digenea, Prosthogonimidae) in *Grus japonensis*

**DOI:** 10.3390/biology13110900

**Published:** 2024-11-05

**Authors:** Yu Cao, Ye Li, Zhong-Yan Gao, Xian-Guang Zhang, Bo-Tao Jiang, Hong-Bao Wang

**Affiliations:** 1 Heilongjiang Academy of Agricultural Sciences Branch of Animal Husbandry and Veterinary Branch, Qiqihar 161005, China; 2 Heilongjiang Provincial Key Laboratory of Veterinary Drugs, Qiqihar 163313, China; 3 Heilongjiang Zhalong National Natural Reserve Administration, Qiqihar 161000, China

**Keywords:** *Grus japonensis*, *Prosthogonimus pellucidus*, ITS sequence, genetic analysis, phylogenetic analyses

## Abstract

Species of the family Prosthogonimidae are considered the most pathogenic trematodes of poultry and wild birds worldwide, causing heavy economic losses in many countries. Prosthogonimosis was a common parasitic disease of *Grus japonensis* (Müller, 1776) which caused inflammation of the cloaca and bursa of Fabricius and even death. Morphological identifications of *Prosthogonimus* species are easily confusing; therefore, molecular characterization is used for discrimination. The present study was conducted to identify *Prosthogonimus* species at Zhalong National Nature Reserve, northeast of China. Considering the morphological variability and wide host range of individual *Prosthogonimus* species, a combination of both morphological and molecular analyses is indispensable for the valid identification of this parasite and the internal transcribed spacer (ITS) region was amplified for the sequence analysis and phylogenetic analysis. The results of molecular analysis together with phylogenetic reconstruction indicated that the *Prosthogonimus pellucidus* (von Linstow, 1873) in this study form a single cluster with *P. pellucidus*, revealing potentially high diversity within the genus *Prosthogonimus*. Classification of *Prosthogonimus* species seems to be unrelated to the host and may be related to geographical location. These data provide a significant resource of molecular markers for studying the taxonomy, population genetics, and systematics of Prosthogonimidae.

## Simple Summary

Species of the family Prosthogonimidae are considered the most pathogenic trematodes of poultry and wild birds worldwide, causing heavy economic losses in many countries. The authors of this study conducted morphological and molecular identification of *Prosthogonimus pellucidus*. These data provide significant molecular markers for the study of taxonomy, population genetics, and systematics of Prosthogonimidae.

## 1. Introduction

**Figure 1:**
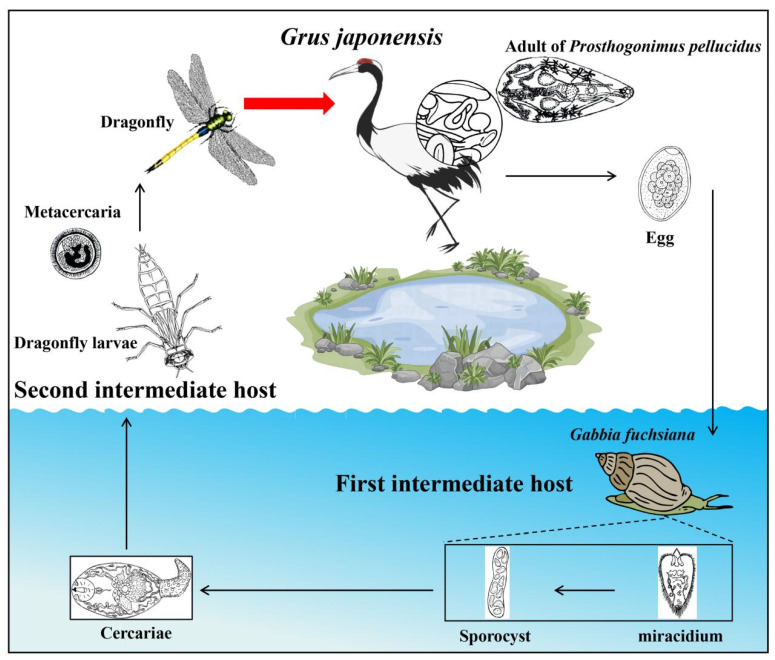
The life cycle of *Prosthogonimus* spp.

Species of the family Prosthogonimidae are considered the most pathogenic poultry trematodes worldwide, affecting particularly free-range poultry and wild birds. They have been reported in many regions worldwide, including Asia, Europe, Africa, and America [1,2,3,4,5]. The *Prosthogonimus* species that have been reported in China include *Prosthogonimus ovatus* (Rudolphi, 1803), *Prosthogonimus cuneatus* (Rudolphi, 1809) and *Prosthogonimus pellucidus* (von Linstow, 1873) [1,6]. *P. pelucidus* is a common *Prosthogonimus* species and is distributed in the south, east, northeast, and western regions of China. The life cycle of *Prosthogonimus* spp. is shown in Figure 1. Qiu and Liu (1983) identified *Gabbia fuchsiana* (Möllendorf, 1888) as the first intermediate host of *P. pellucidus* in China [6]. The snail takes up the trematode eggs deposited in the water and the miracidium that hatched in the intestine of the snail migrates into the hepatopancreas. Here, sporocysts are produced and cercariae are released into the water. Final hosts become infected by dragonflies or damselflies (Odonata), which contain metacercariae of *Prosthogonimus* spp. Adults of *Prosthogonimus* species mainly parasitize in the cloaca, oviduct, and bursa Fabricii of poultry, and excrete eggs in the host’s feces [7]. Poultry infected with *Prosthogonimus* species show inflammation of the oviduct and bursa of Fabricius, and may lay eggs with soft shells or without any shell at all [5]. In addition, *Prosthogonimus* species may rarely also infect mammals, where they reside in the body cavity, intestine, or liver, and lead to peritonitis [8,9]. It has been reported that a nine-month-old infant was infected by *Prosthogonimus* sp. in Indonesia [10].

In spite of the morbidity and economic loss of Prosthogonimosis, there has been an important controversy about the taxonomy of *Prosthogonimus* species [8]. Most studies have mainly focused on the morphology and epidemiology of *Prosthogonimus* species [11,12]. In order to characterize different species in endemic areas, genetic studies of this parasite can be conducted, as genetic methods provide more reliable results. Generally, ITS rDNA sequences show less intra-specific variation than inter-specific variation; therefore, they are considered reliable markers for species differentiation. Some molecular-based studies of internal transcribed spacers could provide valuable genetic markers for species identification of trematodes [13,14,15]. The present research aimed to study ITS2 rDNA sequences as a genetic marker for the genetic characterization of *Prosthogonimus* species, the reconstruction of its phylogenetic relationship, and a comparison of this characterization with previously reported results from *P. pelucidus* obtained from *G. japonicas* and other avian species.

## 2. Materials and Methods

### 2.1. Worms Collection

Adult worms were all collected from the cloaca, oviduct, and bursa Fabricii of naturally infected *G. japonensis* in the Zhalong National Nature Reserve, which followed the wildlife protection law of the People’s Republic of China (a draft of an animal protection law in China released on 2018). A total of 18 trematodes were collected in this study. The worms were washed with normal saline solution and preserved in 70% alcohol at −20 °C.

### 2.2. Morphological Identification

One adult fluke was prepared for morphological studies. For this, the fluke was placed between two glass slides and placed in 70% ethanol for one month. During this month, the force on the slide was gradually increased. The worms were in 50% ethanol and 30% ethanol for 1 h, respectively, then soaked in purified water for 2 h, and stained with hematoxylin solution overnight. The staining solution was poured and purified water, which was used to remove the floating color, and then the worms were put into 30%, 50%, and 70% gradient ethanol, successively. The soaked time of each grade of ethanol was 40 min, 35 min, and 55 min, respectively. Acid alcohol was used for decolorization for 30 s, followed by 80%, 95%, and 100% gradient ethanol. The soaked time of each grade of ethanol was 40 min, 30 min, and 60 min, respectively. The trematode was cleared with xylene for 16 h, embedded in Canada balsam, and examined under a light microscope.

### 2.3. Genomic DNA Extraction and PCR Amplification

Total genomic DNA was extracted from 5 adult worms randomly selected from 18 according to the instructions for the TIANamp Genomic DNA Kit (Tiangen, Beijing, China). The nucleic acid concentration of the extracted DNA was detected, and then the DNA was stored at −20 °C for genetic analysis. The PCR method was used to amplify the ITS sequence of the *Prosthogonimus* species. The PCR reaction was based on previous study primers: NC5: 5′-GTAGGTGAACCTGCGGAACGATCATT-3′, NC2: 5′-TTAGTTTCTTTTCCTCCGCT-3′ [16]. The 25 µL PCR reactions were performed using 18.3 µL of distilled water, 2 µL of dNTP Mixture (2.5 mM), 2.5 µL of 10 × Ex Taq buffer, 0.5 µL of each primer (25 mM), 1 µL of extracted DNA, and 0.2 µL of Ex Taq DNA polymerase (5 U/µL). The DNA template of positive control used fluke DNA stored in the laboratory, previously. The size of the positive control was 1269 bp. The DNA template of negative control used distilled water, under the following conditions: 94 °C for 5 min (initial denaturation), then 94 °C for 30 s (denaturation), 50–65 °C for 1 min (annealing), and 72 °C for 1 min 30 s (extension) for 35 cycles, and a final extension at 72 °C for 10 min.

### 2.4. Sequence Alignments and Phylogenetic Analyses

The positive bands of PCR products amplified by NC2 and NC5 primers were good, which were sequenced using the Sanger method. Sequences were assembled manually and aligned against the fluck of Microphalloidea in GenBank, to identify gene boundaries, using the program DNAStar v. 5.0 [17]. The edited sequence was submitted in GenBank for the accession ID number. The intra-specific variations and inter-specific variation were calculated using MEGA v. 5.0 and MegAlign v. 5.01 [17,18]. Comparisons were made based on nucleotide sequence difference, determined from the ITS2 sequence among *P. cuneatus* (OQ344776.1), *P. ovatus* (KP192735.1), *P. pellucidus* (KP192732.1), and *Prosthogonimus rarus* (Braun, 1901) (KP192728.1). The AT and GC content of the ITS sequences were calculated using DNAStar v. 5.0 [17]. The forward, reverse, complement, and palindromic repeats of the ITS sequences of *P. pellucidus* were examined by REPuter [19]. These repeats were ≥10 bp with a maximum computed repeats of 100 bp.

In addition to our *P. pellucidus* data, the 17-member phylogenetic dataset contains the following: *P. ovatus* (KP192735) from *Passer domesticus* (Poland); *P. ovatus* (KP192733) from *P. domesticus* (Poland); *P. ovatus* (KP192727) from *Anas platyrhynchos* (Czech Republic); *P. ovatus* (KP192723) from *Anas strepera* (Czech Republic); *P. ovatus* (KP192722) from *Aythya ferina* (Czech Republic); *P. ovatus* (KP192730) from *A. platyrhynchos* (Czech Republic); *P. ovatus* (KP192731) from *A. platyrhynchos* (Czech Republic); *P. rarus* (KP192728) from *A. platyrhynchos* (Czech Republic); *P. rarus* (KP192726) from *A. platyrhynchos* (Czech Republic); *P. rarus* (KP192724) from *Anas clypeata* (Czech Republic); *P. cuneatus* (KP192738) from *Turdus merula* (Czech Republic); *P. cuneatus* (KP192736) from *T. merula* (Czech Republic); *P. cuneatus* (KP192729) from *A. platyrhynchos* (Czech Republic); *P. cuneatus* (KP192725) from *A. platyrhynchos* (Czech Republic); *Prosthogonimus falconis* (OK044379) from *Falco peregrinus* (The United Arab Emirates); *P. pellucidus* (KP192734) from *P. domesticus* (Poland); *P. pellucidus* (KP192732) from *A. platyrhynchos* (Czech Republic). And *Collyriclum faba* (JQ231122) from *Saxicola rubetra* was included as an outgroup. Phylogenetic trees were all reconstructed using maximum parsimony (MP) methods. MP methods were performed using the Fitch criterion within PAUP v. 4.0 Beta 10 [20], and bootstrap support values were calculated in PAUP from 1000 bootstrap replicates with 10 random additions per replicate. Phylograms were viewed and drawn using FigTree V. 1.42 [21].

## 3. Results and Discussion

### 3.1. The Morphological Features of P. pellucidus

**Figure 2:**
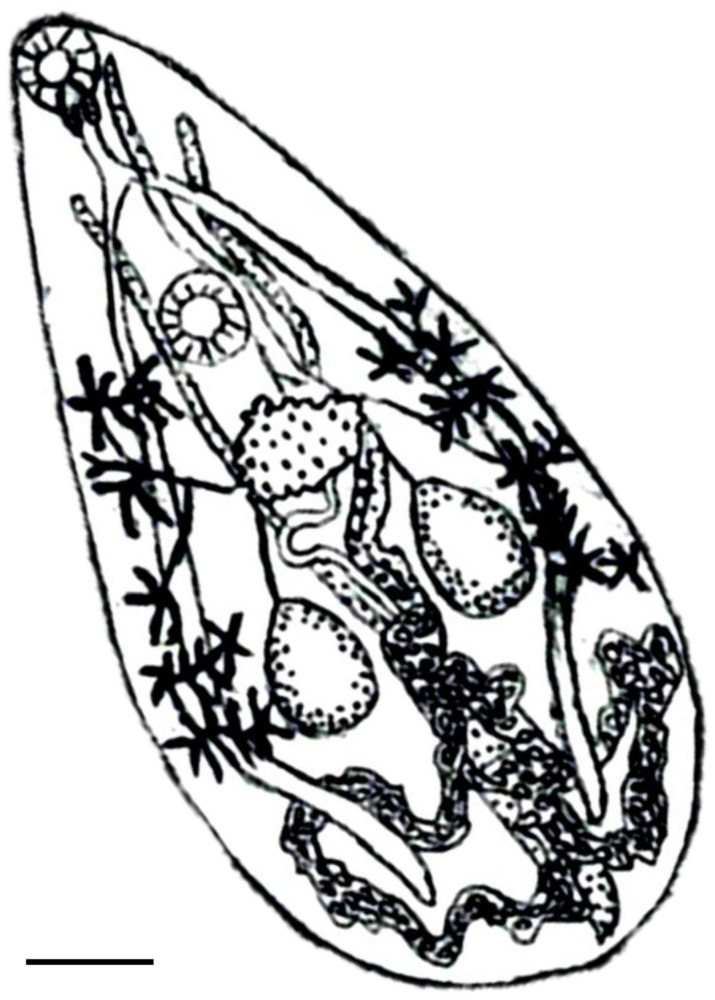
*Prosthogonimus pellucidus*. Scale bar: 0.5 mm.

The examined trematode is pear-shaped, 4.31 mm long with a rounded posterior and a pointed anterior end (Figure 2). The maximum width was 2.5 mm. Minute tegumental spines were observed at the anterior part. The oval ventral sucker (0.61 × 0.64 mm) is situated in the first third of the body. The subterminal oral sucker measures 0.42 × 0.48 mm and is followed by a small spherical pharynx. The two intestinal branches extend nearly to the posterior end. The excretory vesicle is Y-shaped with long branches. The two oval, unlobed testes are situated in a parallel position posterior to the acetabulum in mid-body position and measure 0.58 × 0.35 mm and 0.55 × 0.29 mm. The genital pore is situated lateral to the oral sucker on the anterior end. The deeply lobed ovary is at the posterior edge of the acetabulum. The follicular extra-caecal yolk glands commence from the posterior margin of the acetabulum and extend to the border between second and third portion of the body. The heavily coiled uterus fills the posterior body posterior to acetabulum. A single uterus lobe extends dorsal to the ventral sucker to the genital pore. Operculated eggs in the distal uterus are 27 × 12 µm and contain a fully developed miracidium. According to the above morphological characteristics and the judgment criteria in reference [8,11], it was preliminarily identified as *P. pellucidus*. We attempted to elucidate the morphological characteristics of *P. pellucidus* that might increase our knowledge and understanding of its morphology. Morphological characteristics are generally used to identify adult trematode. However, sometimes morphological parameters are not sufficient as a basis for species differentiation. Thus, molecular approaches are considered for working on the evolution and systematics of trematodes.

### 3.2. The ITS Sequence Features of P. pellucidus

The positive bands of PCR products amplified were good, with a size of approximately 1200 bp, which were sequenced using the Sanger method (Figure S1). Five sequences of the ITS rRNA gene were examined in this study, which were submitted to GenBank. The accession numbers were PP955191, PP958838, PP956936, PP956935, and PP956934, respectively. The lengths of the five partial ITS rDNA sequences obtained were 1183 bp, 1209 bp, 1269 bp, 1261 bp, and 1265 bp, respectively. The ITS sequences contain three genes: internal transcribed spacer-1 (ITS1), 5.8S rDNA-ITS sequence (5.8S), and internal transcribed spacer-2 (ITS2). The lengths of the five partial ITS1 sequences in this study were 852 bp, 854 bp, 900 bp, 901 bp, and 935 bp, respectively. The lengths of the five complete 5.8S sequences in this study were all 107 bp. The lengths of the five partial or complete ITS2 sequences in this study were 224 bp, 227 bp, 248 bp, 254 bp, and 257 bp, respectively.

For 5.8S and ITS2 sequences, the intra-specific variations within *P. pellucidus* in this study were 0%. And the intra-specific variations within *P. pellucidus* in this study of ITS1 sequences was 0–7%. *P. pellucidus* of this study was genetically characterized through ITS rDNA sequences at Zhalong National Nature Reserve, China. Based on the specific location of the study and its only involving one site and one species of avian host, this study had limitations. No separate ITS1 rDNA sequence of *P. pellucidus* was available in the NCBI database. Therefore, the ITS2 rDNA sequence was used for the analysis of Prosthogonimidae sequence differences. Among the five Prosthogonimidae species, the sequence differences in the ITS2 sequence were 2.2−16.0% at the nucleotide level. The *P. ovatus* sequences were the most different from *P. pellucidus* in this study, and these differences were 12.9%. The *P. pellucidus* sequences were the least different from *P. pellucidus* in this study, and these differences were 2.2%.

The five ITS sequences of this study had nucleotide compositions that were biased toward C and G, with an overall C+G content of 50.62–51.94% and A+T content of 48.06–49.38%. The ITS1 sequence nucleotide compositions were biased toward both C and G, with an overall C+G content of 50.82–52.44%, and A+T content of 47.56–49.18%. The 5.8S sequence nucleotide compositions were both biased toward C and G, with an overall C+G content of 51.4% and A+T content of 48.6%. The ITS2 sequence nucleotide composition was biased toward A+T, with an overall A+T content of 49.61–50.89% and C+G content of 49.11–50.39%.

In the current study, forty-two repeat sequences were found in total, and the forward, reverse, complement, and palindromic repeats of the ITS sequences were thirty-four, two, two, and four, respectively. The repeat sequences of ITS1 were the most, with thirty-nine repeat sequences (Table 1). Internal repeats appear to be characteristic of the ITS1 evolution in different groups of organisms [22].

### 3.3. Phylogenetic Analyses

**Figure 3:**
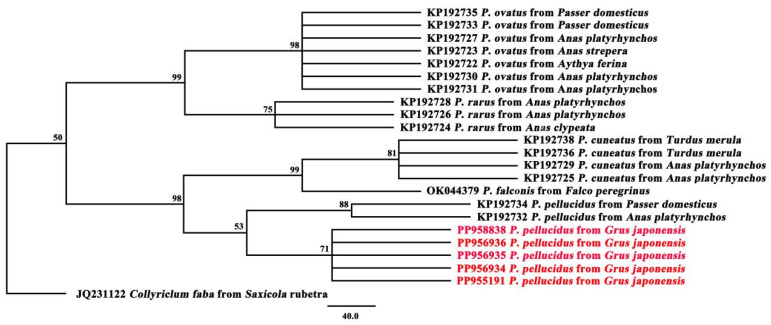
Genetic relationships of *P. pellucidus* with other representative trematodes based on ITS2 sequence data. Phylogenetic analyses used maximum parsimony (MP), with *C. faba* as outgroup. Scale bar indicates posterior probability. The red font represents the trematode in this study.

Phylogenetic analyses of nucleotide sequences from 23 trematode ITS2 sequences were performed using the MP approach (Figure 3). This phylogenetic MP tree splits into two large clades. One clade contains *P. ovatus* and *P. rarus*, and the other clade contains *P. cuneatus*, *P. falconis*., and *P. pellucidus*. In the first clade, different hosts of *P. ovatus* (Poland and Czech Republic) cluster together, and different hosts of *P. rarus* (Czech Republic) cluster together, respectively. In the second clade, different hosts of *P. cuneatus* (Czech Republic) cluster together. *P. pellucidus* from *G*. *japonensis* (China) in this study cluster together, and form a sister taxa with *P. pellucidus* from *P. domesticus* and *A. platyrhynchos* (Poland and Czech Republic). Phylogenetic analyses revealed that classification of *Prosthogonimus* species seems to be unrelated to the host and may be related to geographical location.

The *Prosthogonimus pellucidus* of this study was most closely related to *P. pellucidus* in the previous study, and then to *P. cuneatus*, *P. ovatus*, and *P. rarus.* This is similar to a previous study using concatenated amino acid sequence data representing 12 protein-coding genes, in which *P. cuneatus* and *P. pellucidus* clustered together [23]. This is consistent with a previous study using ITS2, CO1, and ND1 sequences in which *P. pellucidus* and *P. cuneatus* cluster together on one branch, and *P. rarus* and *P. ovatus* cluster together on one branch [24]. In the first clade, *P. ovatus* and *P. rarus* cluster together. It is also consistent with a previous study using ITS2, CO1, and ND1 sequences, in which the phylogenetic relationships of four *Prosthogonimus* spp. were reconstructed and *P. ovatus* and *P. rarus* formed one clade [8]. This is also consistent with a previous study using 28S and CO1 sequences, wherein *P. cuneatus* and cercaria of *P. pellucidus* clustered together [25]. Further characterization of Prosthogonimidae phylogeny will need to wait until additional genomic trematode data have been deposited in GenBank.

## 4. Conclusions

The present study determined the partial sequence of five ITS rDNA sequences of *P. pellucidus* and conducted sequence analysis and phylogenetic analysis. Possibly, the ITS1 marker is a useful genetic marker to study genetic variation with 5.8S and ITS-2 markers, because the ITS1 sequence has repeats and mutation sites, but there are few ITS1 sequences of Prosthogonimidae in the NCBI database; this study added the ITS1 sequence to the database for *P. pellucidus*. Phylogenetic analyses indicate that classification of *Prosthogonimus* species seems to be unrelated to the host and may be related to geographical location, and *P. pellucidus* is the dominant species in this region. From ecological or phylogenetical viewpoints, more detailed genetic analyses will give valuable information, and these data will provide a significant resource regarding molecular markers for studying the taxonomy, population genetics, and systematics of Prosthogonimidae.

## Figures and Tables

**Table 1:** Information of repeat sequences in the ITS rDNA of *Prosthogonimus pellucidus*.

ID	Repeat Start 1	Type	Size (bp)	Repeat Start 2	Repeat Distance	Gene
1	603	Forward	10	668	0	ITS1
2	1066	Forward	10	1179	0	ITS2
3	167	Forward	26	261	0	ITS1
4	167	Forward	26	310	0	ITS1
5	167	Forward	26	359	0	ITS1
6	167	Forward	26	408	0	ITS1
7	167	Forward	26	457	0	ITS1
8	72	Forward	30	306	0	ITS1
9	72	Forward	30	355	0	ITS1
10	72	Forward	30	404	0	ITS1
11	72	Forward	30	453	0	ITS1
12	119	Forward	30	306	0	ITS1
13	119	Forward	30	355	0	ITS1
14	119	Forward	30	404	0	ITS1
15	119	Forward	30	453	0	ITS1
16	123	Forward	30	167	0	ITS1
17	210	Forward	30	306	0	ITS1
18	210	Forward	30	355	0	ITS1
19	210	Forward	30	404	0	ITS1
20	210	Forward	30	453	0	ITS1
21	257	Forward	30	453	0	ITS1
22	257	Forward	35	306	0	ITS1
23	257	Forward	35	355	0	ITS1
24	257	Forward	35	404	0	ITS1
25	347	Forward	38	445	0	ITS1
26	396	Forward	38	445	0	ITS1
27	61	Forward	41	246	0	ITS1
28	293	Forward	43	440	0	ITS1
29	61	Forward	45	108	0	ITS1
30	298	Forward	48	396	0	ITS1
31	167	Forward	73	214	0	ITS1
32	76	Forward	77	167	0	ITS1
33	61	Forward	88	199	0	ITS1
34	298	Forward	97	347	0	ITS1
35	793	Reverse	10	1160	0	ITS1; ITS2
36	638	Reverse	17	638	0	ITS1
37	656	Complement	10	865	0	ITS1
38	576	Complement	11	1151	0	ITS1; ITS2
39	637	Palindromic	10	1048	0	ITS1; ITS2
40	1084	Palindromic	10	1084	0	ITS2
41	1147	Palindromic	10	1147	0	ITS2
42	489	Palindromic	12	489	0	ITS1

## Data Availability

Data are contained within the article and Supplementary Materials.

## Supplementary Materials

The following supporting information can be downloaded at: https://www.mdpi.com/article/10.3390/biology13110900/s1, Figure S1: Agarose gel electropheoresis of ITS PCR products of *P. pellucidus* samples M: maker; 1–5: PCR products of *P. pellucidus* ITS rDNA sequence. 6: positive control; 7: negative control.
