# Supplementary material for: Genetical and Morphological Identification of Prosthogonimus pellucidus (Digenea, Prosthogonimidae) in Grus japonensis
Source: Biology (Basel). 2024 Nov 5;13(11):900. doi: 10.3390/biology13110900 (PMC11591790; doi:10.3390/biology13110900)
Supplement: Supplementary file 1 [file biology-13-00900-s001.zip › biology-3242655-supplementary.pdf]

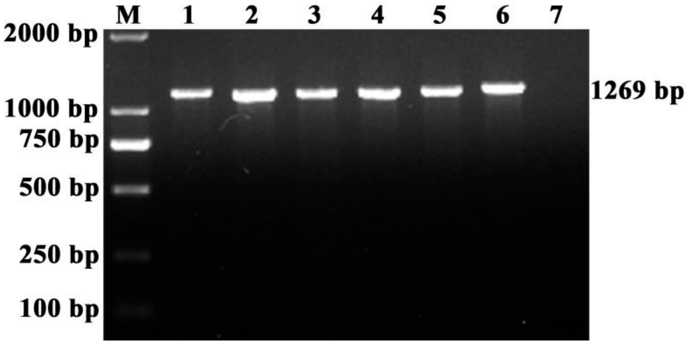

Figure. S1 .Agarose gel electropheoresis of ITS PCR products of *P. pellucidus* samples M: maker; 1-5: PCR products of P. pellucidus ITS rDNA sequence. 6: positive control; 7: negative control.
